# Protein sites with more coevolutionary connections tend to evolve slower, while more variable protein families acquire higher coevolutionary connections

**DOI:** 10.12688/f1000research.11251.2

**Published:** 2017-07-07

**Authors:** Sapan Mandloi, Saikat Chakrabarti

**Affiliations:** 1 Department of Structural Biology and Bioinformatics Division, Council of Scientific and Industrial Research, Indian Institute of Chemical Biology, Kolkata, West Bengal, 700032, India

**Keywords:** Coevolution, evolutionary diversity, correlated mutation, solvent accessibility, cellular localization

## Abstract

*Background*: Amino acid exchanges within proteins sometimes compensate for one another and could therefore be co-evolved. It is essential to investigate the intricate relationship between the extent of coevolution and the evolutionary variability exerted at individual protein sites, as well as the whole protein.

*Methods*: In this study, we have used a reliable set of coevolutionary connections (sites within 10Å spatial distance) and investigated their correlation with the evolutionary diversity within the respective protein sites.

*Results*: Based on our observations, we propose an interesting hypothesis that higher numbers of coevolutionary connections are associated with lesser evolutionary variable protein sites, while higher numbers of the coevolutionary connections can be observed for a protein family that has higher evolutionary variability. Our findings also indicate that highly coevolved sites located in a solvent accessible state tend to be less evolutionary variable. This relationship reverts at the whole protein level where cytoplasmic and extracellular proteins show moderately higher anti-correlation between the number of coevolutionary connections and the average evolutionary conservation of the whole protein.

*Conclusions*: Observations and hypothesis presented in this study provide intriguing insights towards understanding the critical relationship between coevolutionary and evolutionary changes observed within proteins. Our observations encourage further investigation to find out the reasons behind subtle variations in the relationship between coevolutionary connectivity and evolutionary diversity for proteins located at various cellular localizations and/or involved in different molecular-biological functions.

## Introduction

According to the neutral theory of evolution, the functionality of a protein with a disadvantageous mutation can be restored by another mutation that compensates for the first to sustain the function ^1^. Such compensating mutations, together with other factors arising due to common functional, structural and folding constraints, lead to correlations between different positions in a protein or protein family. Coordinated changes of amino acid residues are typically acquired by examining covariation between two aligned positions. A large number of computational methods have been proposed ^2– 11^ to quantify the covariation between two protein sites in a given multiple sequence alignment (MSA). Most methods are based on variation of mutual information ^12– 17^, maximum likelihood approximations ^18^, Bayesian probabilities ^19^, and phylogenetic approaches ^20, 21^. Newer methods successfully implement direct coupling analysis ^22^, Protein Sparse Inverse COVariance: PSICOV ^23^ and Matrix Match Maker ^24^ algorithms to identify coevolving sites. These previous studies demonstrate that sequence covariation is powerful in detecting protein-protein interactions, ligand receptor binding, and the folding structure of a protein. In addition to direct physical interactions, distantly located coevolving amino acid residues are reported to be energetically coupled ^25^ or subject to similar functional constraints ^26^. Compensated amino acid substitutions have been described in previous works in terms of their locations in structure and their physico-chemical properties ^3, 20, 21^. Coevolutionary signals coming from residue charge compensating mutations have been found to be stronger compared to size compensating mutations ^3, 21, 27^. Despite the fact that coevolution has been found to be rather weak in many cases, correlated mutations have had comparative success in predicting protein secondary and tertiary structures, and in some cases protein interaction partners ^28– 30^.

Coevolution is difficult to detect due to various reasons, such as the variable nature of compensatory mutations, the strong dependence of covariations on evolutionary distances, and the number of sequences in the alignment. Hence, it is crucial to understand how coevolutionary processes are related to evolutionary diversity within protein families. Despite significant efforts in this field, the relationship between evolutionary conservation and the extent of coevolution is not well understood. For example, it is not clear whether families with higher evolutionary diversity would exhibit more coevolutionary connections or not. Similarly, at the residue level, this relationship needs to be thoroughly examined. An earlier study by Fodor and Aldrich ^31^ observed a lack of agreement between correlated mutation methods, and the resultant differences might have been caused by differing sensitivities to background conservation. In a previous study, it was also indicated that residues, which form many coevolutionary connections with other residues, are more evolutionary conserved and are involved in specific functionally important interactions and conformational changes ^32^.

A complete understanding of protein evolution and coevolution will require a large scale analysis of important factors that determine the selective forces acting on different residues of a protein to be coevolved. Here, we present a study that undertook a detailed analysis to investigate the relationship between evolutionary conservation and the extent of coevolution within a protein. This relationship could be dependent on the reliability of the predicted coevolved sites as there are no direct ways to validate the coevolutionary connectivity. Therefore, it is a good idea to use multiple coevolution extracting algorithms and filter out a reliable set within protein sites. Similarly, spatial proximity between the coevolved sites might provide additional reliability about the predicted coevolved sites ^33^. We examined the evolutionary conservation using the popular AL2CO ^34^ program within 19,736, 35,514, 50,217, and 56,879 coevolved site pairs (located within 10Å spatial distance), which were identified by approaches, such as mutual information (MIp program ^6^), McLachlan amino acid similarity matrix based techniques (McBASC program ^27^), Direct Coupling Analysis (DCA program ^22^), and Protein Sparse Inverse COVariance method (PSICOV program ^23^), from 753 curated protein family alignments, available from the Conserved Domain Database (CDD ^35^). Our study suggests the hypothesis that a higher number of coevolutionary connection is likely to be observed for a particular site that is less evolutionary variable, while a higher number of coevolutionary connections can be observed for a protein family that has higher evolutionary variability. We found that the sites with a higher number of coevolutionary connections have a much higher tendency to be conserved compared to the sites with a smaller number of connections. These sites might act as ‘hub points’, and therefore changes in these sites would affect many other connected sites. We further investigated the impact of important structural properties, like secondary structures, solvent accessibilities and hydrogen bonding of the coevolved sites, to understand the reasons behind the observed correlation between coevolution and evolutionary diversity. Our findings indicate that coevolved sites are generally preferred at a solvent accessible/hydrogen bonded/helical state compared to a solvent buried/non-hydrogen bonded/β strand state. However, discernable differences in evolutionary conservation between the higher and lesser coevolved sites were observed only for sites located at solvent accessible states compared to buried states. We also examined whether the observed negative (anti) correlation between coevolution and evolutionary conservation for a protein family can be under the influence of its cellular localization or the type of functions with which it is involved. Coevolution analysis for the whole protein suggests that the cytoplasmic and extracellular proteins possess moderately stronger negative (anti) correlation between the number of coevolutionary connections and their average evolutionary conservation.

## Methods

### Dataset

We collected 753 protein domain alignments from the Conserved Domain Database (CDD; https://www.ncbi.nlm.nih.gov/Structure/cdd/cdd.shtml ^35^) version 2.13, for which at least one 3D structure entry and more than 50 protein sequences are available. An alignment length threshold (>=100) was also applied to exclude smaller proteins. A complete list of protein families is provided in Table S1.

### Identification of coevolved sites

Mutual information ^6^ (Suppl. Mat. Ref.) is widely used measure to estimate the covariation between sites in protein families. In this analysis, we used a mutual information based method to estimate coevolutionary connection between two sites of a protein family. This method (MIp) is based on information theory that accurately estimates the expected levels of background coming from random and phylogenetic signals. Removal of the phylogenetic and random background allows identifying substantially more coevolving positions in protein families. Altogether we identified 19,736 (out of total 36,616) coevolved site pairs located within 10Å spatial distance from the 753 family alignments, with a MIp Z-score cutoff of 4.0 or higher.

McBASC ^27^ ( http://fodorwebsite.appspot.com//covariance1_1.zip) was used to calculate the simple inter-position coevolution for the 753 protein family alignments. McBASC provides high score for non-conserved and co-varying positions from a multiple sequence alignment. The calculation of McBASC was performed as described in Fodor and Aldrich 2004, using the software provided by the authors ( http://www.afodor.net/). McBASC does not use any structural or phylogenetic information in the calculation of coevolution. We identified 35,514 (out of total 95,866) coevolved site pairs located within 10Å spatial distance from the 753 family alignments with McBASC Z-score cutoff of 4.0 or higher.

DCA ^22^ (Direct Coupling Analysis) aims at predicting coevolving residues based on the maximum entropy principle. DCA is also used in predicting inter and intra domain contacts. This method is used in separating direct and indirect correlation between residues. DCA analysis was implemented with MATLAB code kindly provided to us by Domenico L. Gatti ( Supplementary File 1). We identified 50,217 (out of total 1,61,332) coevolved site pairs located within 10Å spatial distance from the 753 family alignments with DCA Z-score cutoff of 4.0 or higher.

PSICOV ^23^ (Protein Sparse Inverse COVariance) method is developed with the specific goal of separating direct from indirect coupling between residues. PSICOV takes into account the global correlations between pairs. Modified MATLAB code (without the default minimum requirement of 500 sequences), which was kindly provided to us by Domenico L. Gatti ( Supplementary File 1), was used in this study. We identified 56,879 (out of total 162,336) coevolved site pairs located within 10Å spatial distance from the 753 family alignments with PSICOV Z-score cutoff of 4.0 or higher.

### Random selection of non-coevolved sites and pairs

Site pairs other than those involved in coevolutionary connections were considered as non-coevolutionary sites. We randomly selected non-coevolved sites from each protein family ( Supplementary File 2). For each randomly selected non-coevolved site (i), neighboring non-coevolved sites were selected based on the structural distance (<10Å) and sequence distance filters (>i±6 positions). Similar numbers of non-coevolved site pairs were selected randomly 10 times. We performed similar correlation analysis between the numbers of spatial neighbors and evolutionary conservation of non-coevolved sites.

### Calculation of amino acid conservation

Analysis of positional conservation in a sequence alignment can aid in the detection of functionally and/or structurally important residues. The AL2CO ^34^ program performs conservation analysis in a comprehensive and systematic way. It was used to calculate the conservation index for each position for a given multiple sequence alignment. Twelve different strategies of conservation index calculation have been implemented in the AL2CO program ( http://prodata.swmed.edu/al2co/al2co.php). For this analysis, we used independent count (sequence weighting scheme) and matrix based sum-of-pair ^36^ (conservation calculation method) measure scoring scheme to calculate evolutionary conservation of each coevolved sites or column in the alignment. A higher AL2CO score indicates higher conservation index.

### Calculation of spatial distances and structural properties

Representative three-dimensional (3D) structures were collected for each family from the Protein Data Bank (PDB; http://www.rcsb.org/pdb/home/home.do) ^37^. Spatial distances were calculated using atom coordinates supplied in the individual PDB file. Structural properties, such as solvent accessibility, secondary structures, and hydrogen bonds, were computed from the protein structure using the JOY package ^38 ^( http://mizuguchilab.org/joy/) Solvent accessibility was measured using the PSA program from the JOY package, and residues that had an accessible surface area <7% were treated as solvent buried or inaccessible. Similarly, secondary structures (helix, strand and coil) and hydrogen bonding patterns were estimated using the SSTRUC and HBOND programs from the JOY package ^39^, respectively.

### Collection of Gene Ontology information

The Gene Ontology ( http://www.geneontology.org/) ^40^ covers three classes/domains: cellular localization, molecular function and biological process. Functional information of each CDD family was collected from Gene Ontology database using the UNIPROT ^39^ ID of the representative protein structure as a query. We mapped 517, 720, and 634 protein domain families into cellular localization, molecular function and biological process, respectively.

### Mapping conservation and coevolutionary connection onto 3D structure

Mapping of evolutionary conservation and coevolutionary information onto the 3D structure was done using in-house Perl scripts ( Supplementary File 3). B-Factor column in PDB file was substituted with evolutionary conservation score and colored according to B-Factor ranging blue (low conservation) to red (high conservation). Lines connecting C-alpha atoms of residues represent coevolutionary connection between those residues.

## Results and discussion

### Coevolution versus evolutionary diversity at the site level

Coevolutionary connections between protein sites were identified from multiple sequence alignments of 753 protein domain families by algorithms employing differing approaches, such as mutual information (MIp program ^6^), McLachlan amino acid similarity matrix based techniques (McBASC program ^27^), Direct Coupling Analysis (DCA program ^22^), and Protein Sparse Inverse COVariance method (PSICOV program ^23^). Minimal overlaps were observed for coevolved sites predicted by these programs ( Figure S1), supporting previous interpretations that differences in the preferred level of background conservation may exist within each program to identify coevolved residue pairs ^6^.

**Figure 1.**
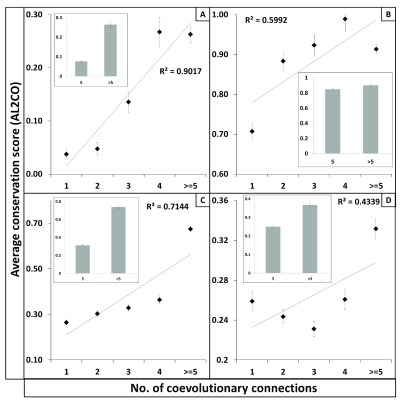
Relationship between coevolutionary connections and evolutionary conservation for protein sites. X-axes show the coevolutionary connection (represented in bins of 1 and 5) per site whereas Y-axes represent the average evolutionary conservation index (CI) estimated by the AL2CO program. Each vertical panel (panel **A**– **D**) represents results obtained from coevolution predicted by various programs (Panel **A**: MIp; **B**: McBASC; **C**: DCA; **D**: PSICOV). Panels provide correlation data for the coevolved sites that are located within or equal to 10Å. The coefficient of determination (R ^2^) indicates how well the data points fit to the linear regression model between coevolutionary connection and evolutionary conservation. The observed scale of coevolution values obtained from multiple covevolutionary programs varies a lot. The probable reason for such observation can be the algorithm used by individual programs for calculation of covariation/coevolution.

The pattern of evolutionary diversity within the coevolved sites was examined using evolutionary conservation scoring approaches ( *e.g*., AL2CO). Figure 1 plots the average conservation scores of sites having higher or lower coevolutionary connections (A: MIp; B: McBASC; C: DCA; D: PSICOV programs, respectively). Figure 1 suggests that highly coevolved sites possess higher average AL2CO scores, depicting higher evolutionary conservation. Coevolutionary connections, even though selected based on a strong statistically significant threshold (Z-score >4), might contain background noise resulting in an unreliable relationship between coevolution and evolutionary conservation. To disprove this, we performed similar analysis using non-coevolutionary random sites and found that there is a smaller correlation between non-coevolved sites having higher or lower structural distance based neighbors (<10Å) and their evolutionary conservation ( Figure S2).

Observation of strong positive correlation between coevolutionary connections and evolutionary conservation within the coevolved sites selected based on structural proximity suggests that highly coevolved protein sites tend to evolve slower.

***Influence of structural environment.*** The structural environment of a protein site is a critical factor that can influence its evolutionary diversity pattern ^41, 42^. To understand the reasons behind the observed phenomenon where higher coevolutionary connections are found for sites that are less diversified, we investigated the roles of structural environments, such as solvent accessibility state, and secondary structural content of the coevolved sites.

**Figure 2.**
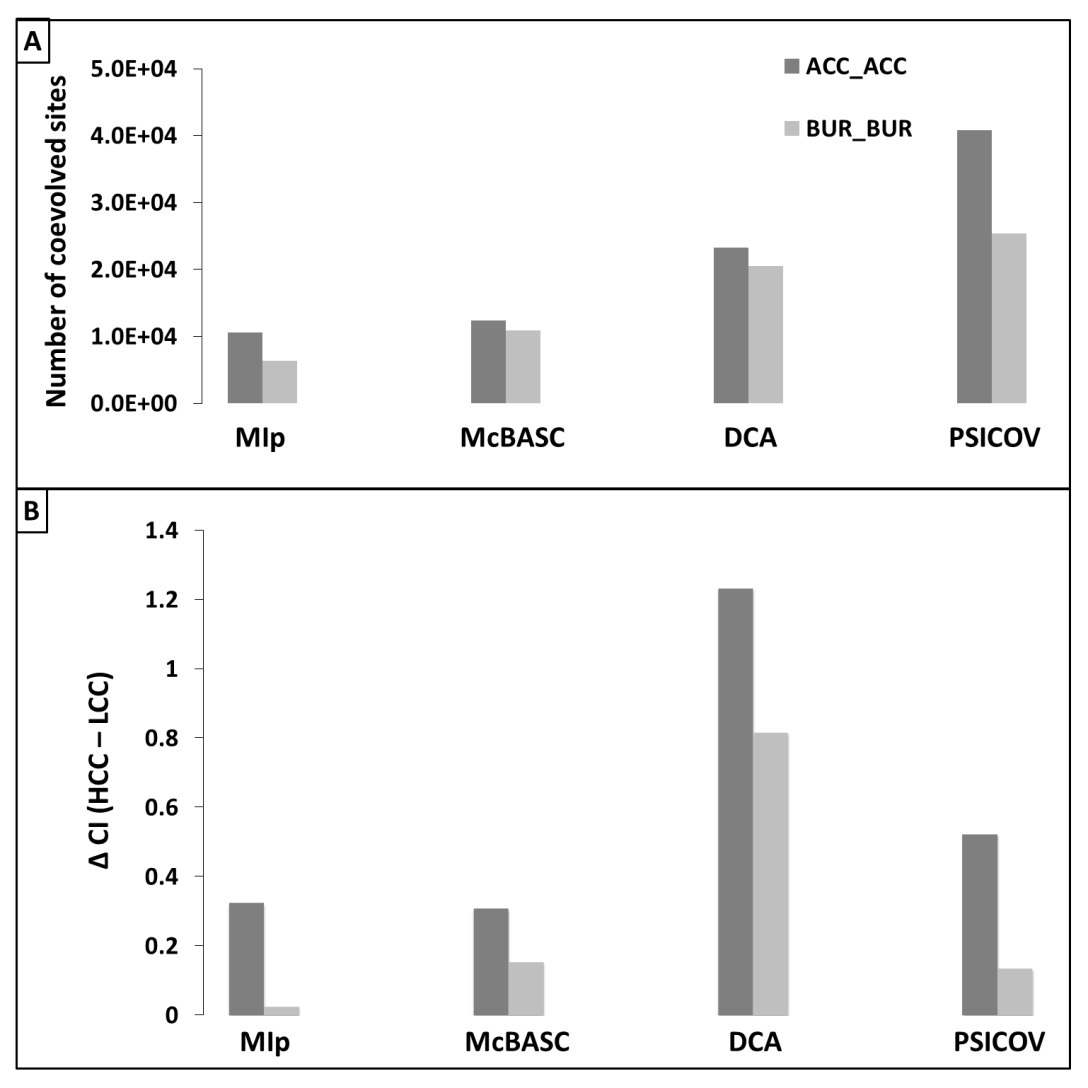
Analysis for sites involved in solvent accessible and buried environment. ( **A**) Number of coevolved sites involved in forming coevolutionary pairs, where both sites are present in solvent accessible (ACC_ACC; dark grey) and buried (BUR_BUR; light grey) environments. ( **B**) Difference of conversation indices (CI) between higher coevolutionary connection (HCC) and lower coevolutionary connection (LCC) sites involved in ACC_ACC and BUR_BUR environments. LCC: less than or equal to 3 coevolutionary connections; HCC: higher than 3 coevolutionary connections.

We have observed more coevolutionary connections for sites that are solvent accessible compared to that observed within buried sites ( Figure 2A). Interestingly, solvent accessible sites that possess lower numbers (<3) of coevolutionary connections (LCC) are consistently less conserved compared to the sites that have relatively higher number (>3) of coevolutionary connections (HCC) ( Figure 2A). Although the similar trend is also observed within the solvent buried sites, the differences of conversation indices between the HCC and LCC are more prominent within solvent accessible state compared to that observed at buried state ( Figure 2B).

Higher abundance of coevolutionary connections is also observed for sites that are involved in hydrogen bonding compared to those are not involved in hydrogen bonding. However, no discernable differences in evolutionary conservation were observed between the higher and lesser coevolved sites involved in hydrogen bonding compared to those that do not have hydrogen bonding ( Figure S3).

Slightly higher abundance of coevolutionary connections was observed for sites that were located in helix compared to those forming strands. No discernable differences in evolutionary conservation were observed between the higher and lesser coevolved sites located at helical environments compared to those that form strands ( Figure S4).

***Influence of functional involvement.*** We also investigated the relationship between coevolutionary connection and evolutionary conservation for protein sites with respect to their functional involvement. However, functional sites (e.g., active sites, protein or ligand binding sites) do not show significantly higher positive correlation between coevolutionary connection and evolutionary conservation, and no discernable differences were observed among the correlation coefficients between coevolutionary connection and evolutionary conservation observed for various types of functional sites (data not visualised).

### Coevolution versus evolutionary diversity at the protein/family level

**Figure 3.**
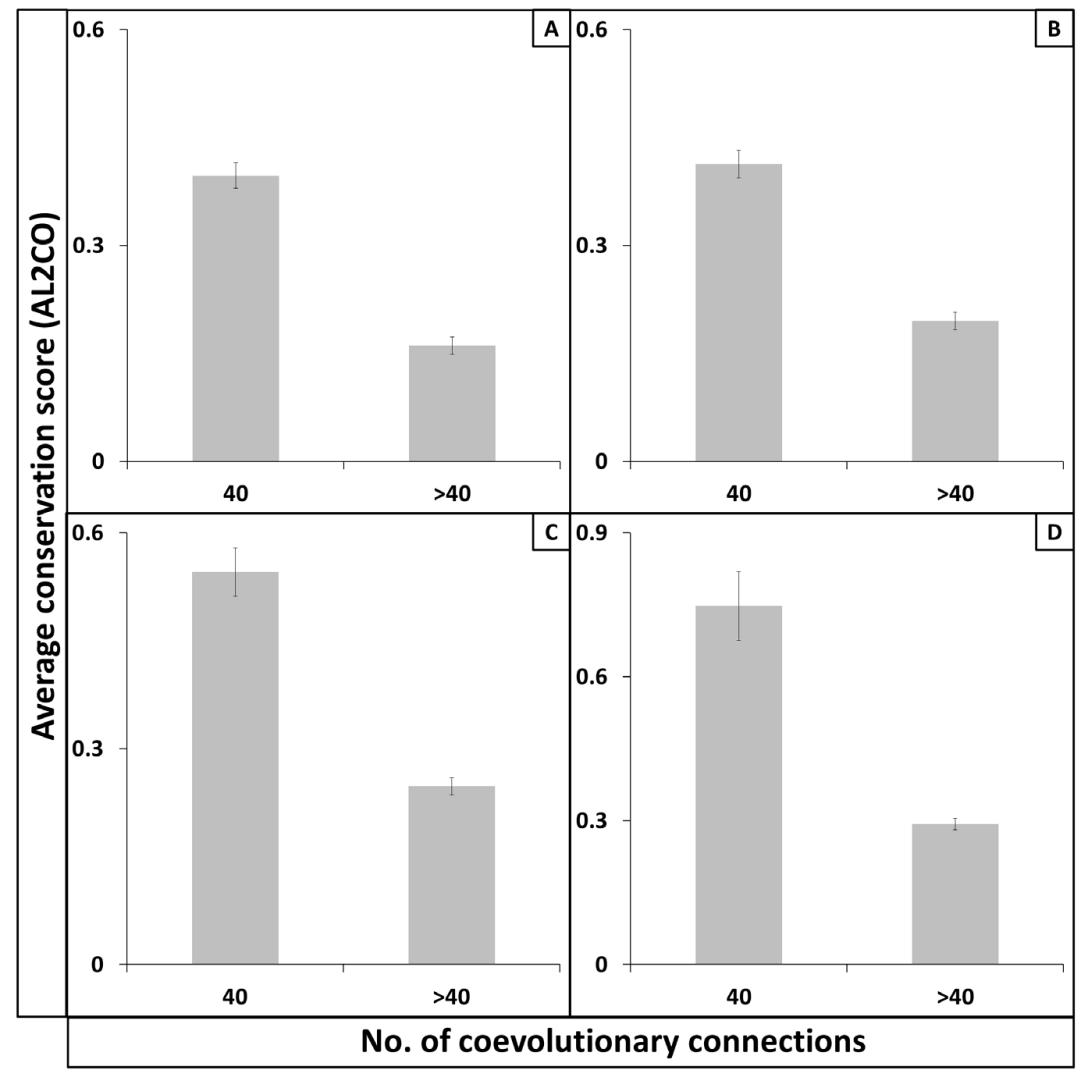
Relationship between coevolutionary connections and evolutionary conservation for the full-length protein. X-axes show the coevolutionary connections (represented in bins of 40) of protein families whereas Y-axes represent the average evolutionary conservation score of the same families estimated by the AL2CO program. Panels show the data extracted from all 753 CDD families. Each panel ( **A**– **D**) represents results obtained from coevolution predicted by various programs (MIp, McBASC, DCA and PSICOV, respectively).

It is important to know how the evolutionary conservation profile of the whole protein or family influences the coevolutionary connections within its sites. Figure 3 and Figure S5 plot the average conservation scores of protein families (considering all gapless columns of the family alignment) with respect to the total number of coevolved sites observed within those families. Our results suggest strong negative correlation between the number of coevolved sites found within a protein family and its average conservation score. This finding indicates that, in general, more conserved proteins/families tend to possess lower coevolutionary connections, whereas proteins/families with less stringent evolutionary pressure might engineer more intra-coevolutionary connections.

We further investigated the influence of cellular localization and biological-molecular functions of the proteins that displayed correlation between the coevolutionary connections and evolutionary conservation. We categorized the representative proteins from 517, 720, 634 families into cellular localization, molecular function and biological processes, respectively, using their Gene Ontology annotations. For example, 54%, 15% and 12% of the 517 families, having at least one pair of coevolved sites, reside within cytoplasm, nucleus and membrane, respectively ( Figure S6). Similarly, 55%, 17% and 10% coevolved protein families are involved in catalysis (enzyme), nucleic acid and ion binding functions, respectively. Coevolved proteins were also found to be abundant in various metabolic functions ( Figure S6). Table 1 provides the R ^2^ and slope (m) values between the coevolutionary connection and evolutionary conservation for proteins categorized in certain cellular localization. Cytoplasmic and extracellular proteins show slightly stronger anti-correlation between the number of their coevolutionary connections and evolutionary conservation. Similarly, proteins involved in catalysis and nucleic acid binding type of molecular functions show moderately stronger negative correlation, whereas proteins involved in miscellaneous metabolic processes, which mostly include generic carbohydrate and glutamine metabolisms and nitrogen fixation processes, exhibit stronger negative correlation between coevolutionary connections within the protein and its average conservation ( Table 1).

**Table 1.** Correlation between the coevolutionary connections and the evolutionary conservation of proteins with respect to their Gene Ontology classification. R ^2^: coefficient of determination; m: slope of line for relationship between the coevolutionary connections (predicted by MI, McBASC, DCA and PSICOV programs) and the evolutionary conservation of proteins with respect to their most frequently observed Gene Ontology based cellular localizations, molecular functions, and biological processes.

	Gene Ontology											
Program	Cellular localizations				Molecular functions				Biological processes			
	Cytoplasm	Nucleus	Membrane	Extracellular space	Catalysis	Nucleic acid binding	Ion binding	Ligand binding	Anabolic processes	Catabolic processes	Other metabolic processes	Transport
**MIp**	**R² = 0.63**	**R² = 0.12**	**R² = 0.6**	**R² = 0.70**	**R² = 0.82**	**R² = 0.43**	**R² = 0.51**	**R² = 0.40**	**R² = 0.84**	**R² = 0.62**	**R² = 0.77**	**R² = 0.54**
	**m = -0.03**	**m = -0.03**	**m = -0.06**	**m = -0.06**	**m = -0.05**	**m = -0.05**	**m = -0.05**	**m = -0.02**	**m = -0.01**	**m = -0.02**	**m = -0.04**	**m = -0.06**
**MCBASC**	**R² = 0.68**	**R² = 0.62**	**R² = 0.2**	**R² = 0.53**	**R² = 0.55**	**R² = 0.53**	**R² = 0.55**	**R² = 0.25**	**R² = 0.15**	**R² = 0.40**	**R² = 0.51**	**R² = 0.24**
	**m = -0.04**	**m = -0.07**	**m = -0.03**	**m = -0.06**	**m = -0.08**	**m = -0.06**	**m = -0.05**	**m = -0.02**	**m = -0.01**	**m = -0.04**	**m = -0.04**	**m = -0.05**
**DCA**	**R² = 0.93**	**R² = 0.47**	**R² = 0.54**	**R² = 0.37**	**R² = 0.62**	**R² = 0.83**	**R² = 0.63**	**R² = 0.68**	**R² = 0.27**	**R² = 0.54**	**R² = 0.81**	**R² = 0.48**
	**m = -0.06**	**m = -0.09**	**m = -0.08**	**m = -0.05**	**m = -0.10**	**m = -0.07**	**m = -0.07**	**m = -0.04**	**m = -0.02**	**m = -0.14**	**m = -0.07**	**m = -0.09**
**PSICOV**	**R² = 0.41**	**R² = 0.01**	**R² = 0.42**	**R² = 0.61**	**R² = 0.74**	**R² = 0.67**	**R² = 0.4**	**R² = 0.35**	**R² = 0.16**	**R² = 0.43**	**R² = 0.89**	**R² = 0.0006**
	**m = -0.05**	**m = -0.01**	**m = -0.05**	**m = -0.10**	**m = -0.27**	**m = -0.10**	**m = -0.04**	**m = -0.04**	**m = 0.01**	**m = -0.24**	**m = -0.07**	**m = -0.01**

### Example cases

**Figure 4.**
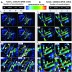
Coevolutionary connection and evolutionary conservation are projected onto the 3D structures of proteins from two different protein families. Panel **A1** provides an example of higher coevolutionary connections (average >20) with respect to an overall lower evolutionary conservation (average AL2CO score: 0.65) status projected on the 3D structure of a representative protein (PDB code: 1DJ0) from the pseudouridine synthase domain family (CDD code: CD01291). Panel **B1** represents a case [representative protein (PDB code: 1SRO; CDD code: CD00164) from the ribosomal protein S1 domain] where lower coevolutionary connections (average <10), with respect to overall higher evolutionary conservation (average AL2CO score: 1.63) status are observed. Lower panels ( **A2** and **B2**) show examples from the same families (zoomed image) of higher coevolutionary connections for sites that have relatively higher evolutionary conservation.

Figure 4A provides an example case where coevolutionary connections are overlaid with evolutionary conservation scores onto the 3D structure of a representative protein (PDB code: 1DJ0) from the pseudouridine synthase domain family (CDD code: CD01291). 8, 30, 20 and 46 coevolutionary connections were predicted by MIp ^6^, McBASC ^27^, DCA ^22^ and PSICOV23 methods, respectively. Interestingly, in this family, the average conservation score (AL2CO score: 0.65) for all sites are quite low (as shown by color coding), despite having higher coevolutionary connections. Hence, observations in this family support the hypothesis that a higher number of coevolutionary connections can be expected for a protein family that has higher evolutionary variability or lower evolutionary conservation. Similarly, Figure 4B provides a case where coevolutionary connections are projected onto the 3D structure of a representative protein (PDB code: 1SRO) from the ribosomal protein S1 domain (CDD code: CD00164). It is evident from Figure 4B that the number of coevolutionary connections is relatively low in this family, while the overall evolutionary conservation (indicated by color coding) is higher (AL2CO score: 1.63). Hence, observations in this protein support the hypothesis that lower evolutionary connections can be expected for a less evolutionary variable protein. Interestingly, sites within the 1SRO protein show a similar trend as observed in the 1DJ0 protein (panels A2 and B2 of Figure 4), and higher numbers of coevolutionary connections are observed for protein sites that are less evolutionary variable.

Predicted data for coevolution and conservation Files of coevolutionary sites predicted by four programs with conservation score predicted by AL2CO program with 10Å filter. Click here for additional data file. Copyright: © 2017 Mandloi S and Chakrabarti S 2017 Data associated with the article are available under the terms of the Creative Commons Zero "No rights reserved" data waiver (CC0 1.0 Public domain dedication).

In order to compare our findings, we have performed a similar analysis using the MISTIC ^43^ (Mutual Information Server To Infer Coevolution) server, taking 20 randomly selected protein families from our dataset as case study. Interestingly, the observed coevolutionary network predicted for CD01291 and CD00164 families (discussed above) are similar to our study ( Figure S7). MISTIC results also show that the CD01291 family has higher coevolutionary network connections for fewer variables sites whereas the CD00164 family has less coevolutionary connections and is overall less conserved. The MISTIC server’s web link results for other protein families, are available in Table S2.

## Conclusions

Over the years, it has become apparent that intra protein coevolution is an important evolutionary phenomenon to maintain proteins’ functional flexibility. However, the signs of coevolution are subtle, and as a consequence, hard to detect. The majority of sites in a protein coevolve to some degree, in that they contribute more or less to structural integrity and, thus, function of the protein. However, some sites will more directly influence each other. By definition, coevolution is closely connected to the evolutionary variability of a protein. Hence, it is essential to investigate the intricate relationship between the extent of coevolution and the evolutionary variability exerted at individual protein sites, as well as the whole protein. However, it is also relevant to check the reliability of the predicted coevolved sites before deriving any hypothesis between coevolution and evolutionary conservation. Therefore, we employed multiple algorithms for the detection of coevolutionary connection and used a structural proximity based filtration system to validate the coevolutionary connections within protein sites.

In this study we have not checked/compared the difference between the two concepts of covariation and coevolution. We have used different programs (MIp, McBASC, DCA and PSICOV) which calculate covariation among protein sites in tree-independent manner. In this study, it was assumed that observed patterns of covariation are caused by molecular coevolution and they were treated synonymously. To the best of our knowledge, this is the first time where such a detailed analysis is performed to investigate any existing correlation between the coevolution and evolutionary conservation. Based on our observations, we propose an interesting hypothesis that a higher number of coevolutionary connection is associated for a protein site that is less evolutionary variable, while a higher number of the coevolutionary connections can be observed for a protein family that has higher evolutionary variability. The obvious question is why such apparently contrasting relationship exists. One probable explanation could be that these highly coevolved sites might act as ‘coevolutionary hubs’, and therefore changes at these sites would affect many other connected sites. On the contrary, the evolutionary selection pressure needs to be lower at the whole protein for more sites to be involved in covariation. Probably, sites that are critical to maintain structural integrity and functional flexibility are co-varying with many other sites, but the extent of variation is limited. Hence, the critical balance between covariation and evolutionary conservation is maintained via these ‘coevolutionary hub’ sites. However, to be rich in a coevolutionary connection, a protein requires evolutionary flexibility so that correlated or compensatory mutations can be arranged with response to an initial change. Hence, higher coevolutionary connection is observed for families that are more evolutionary variable than others.

## Data availability

The data referenced by this article are under copyright with the following copyright statement: Copyright: © 2017 Mandloi S and Chakrabarti S

Data associated with the article are available under the terms of the Creative Commons Zero "No rights reserved" data waiver (CC0 1.0 Public domain dedication). 

**Dataset 1: Predicted data for coevolution and conservation.** Files of coevolutionary sites predicted by four programs with conservation score predicted by AL2CO program with 10Å filter. doi, 10.5256/f1000research.11251.d157108 ^44^

## Supplementary material

**Table S1: List of CDD families.** File contains CDD family ID, PDB ID and UNIPROT ID used in the study.

**Table S2: MISTIC server results for CDD protein families.**

**Supplementary File 1: Program for coevolutionary connection prediction.** MATLAB code for DCA and PSICOV coevolutionary connection prediction program. This code is provided by Domenico L. Gatti with permission.

**Supplementary File 2: Program to extract non-coevolved sites.**

**Supplementary File 3: Program and data-files to map conservation and coevolutionary information onto PDB.**

**Figure S1: Number of coevolved pairs predicted (in blue, pink, green and yellow) by different programs (MIp, MCBASC, DCA and PSICOV, respectively) and common pairs (in black) between them.**

**Figure S2: Relationship between the number of structural neighbor and evolutionary conservation for non-coevolved protein sites.** X-axes show the numbers of structural neighbor (represented in bins of 1) per site whereas Y-axes represent the average evolutionary conservation index estimated by the AL2CO program. Each panel (panel **A**– **D**) represents results obtained from the number of non-coevolved pairs similar to the number of coevolved pairs predicted by various coevolution programs (A: MIp; B: McBASC; C: DCA; D: PSICOV). Panels provide correlation data for the non-coevolved sites (i) that are located within or equal to 10Å (and at sequence position between i and >i±6). The coefficient of determination (R ^2^) indicates how well the data points fit to the linear regression model between coevolutionary connection and evolutionary conservation.

**Figure S3: Analysis for sites involved in H-bonding.** ( **A**) Number of coevolved sites involved in forming coevolutionary pairs, where both sites are either involved in H-bonding (HBY_HBY; dark grey) or not involved in H-bonding (HBX_HBX; light grey). ( **B**) Difference of conversation indices (CI) between higher coevolutionary connection (HCC) and lower coevolutionary connection (LCC) sites involved in HBY_HBY or HBX_HBX. LCC: less than or equal to 3 coevolutionary connections; HCC: higher than 3 coevolutionary connections.

**Figure S4: Analysis for sites involved in different secondary structures.** ( **A**) Number of coevolved sites involved in forming coevolutionary pairs, where both sites are located in helix (H_H; dark grey) and strand (E_E; light grey). ( **B**) Difference of conversation indices (CI) between higher coevolutionary connection (HCC) and lower coevolutionary connection (LCC) sites involved in H_H and E_E. LCC: less than or equal to 3 coevolutionary connections; HCC: higher than 3 coevolutionary connections.

**Figure S5: Relationship between coevolutionary connections and evolutionary conservation for the full-length protein.** X-axes show the coevolutionary connection (represented in bins of 10) of protein families whereas Y-axes represent the average evolutionary conservation score of the same families estimated by the AL2CO program. Panels show the data extracted from all 753 CDD families. Each panel ( **A**– **D**) represents results obtained from coevolution predicted by various programs (MIp, MCBASC, DCA and PSICOV, respectively).

**Figure S6: Gene Ontology distribution of the protein families used for this study.** ( **1**) Representative proteins from 517 CDD families were assigned to cellular localization, whereas the same from 720 and 624 families could be assigned to at least one ( **2**) molecular function or ( **3**) biological process ( **3**), respectively. Details can be found in Methods.

**Figure S7: MISTIC server results as Circos representation for (A) CD01291 and (B) CD00164 families.** MI Circos is a sequential circular representation of the MSA and the information it contains. Colored square boxes of the second circle indicate the MSA position conservation (highly conserverd positions are shown in red, while less conserved ones are shown in blue). Lines connect pairs of positions with MI greater than 6.5 ( Marino Buslje *et al.*, 2009). **Red** edges represent the top 5%, **black** ones are between 70% and 95%, and **gray** edges account for the remaining 70%.
