# Supplementary material for: Protein sites with more coevolutionary connections tend to evolve slower, while more variable protein families acquire higher coevolutionary connections
Source: F1000Res. 2017 Jul 7;6:453. Originally published 2017 Apr 10. [Version 2] doi: 10.12688/f1000research.11251.2 (PMC5506539; doi:10.12688/f1000research.11251.2)
Supplement: Supplementary file 3 [file f1000research-6-13045-s0002.tgz › a9675696-a66f-4f6d-928e-efab1217353a.pdf]

Table S2: MISTIC Server result for CDD protein families.

CDD code	Family name	Result URL
CD01424	MGS_CPS_II	http://mistic.leloir.org.ar/results.php?jobid=20170611337071057
CD01887	Initiation Factor 2 (IF2)	http://mistic.leloir.org.ar/results.php?jobid=20170611409092676
CD03377	Thiamine pyrophosphate (TPP family)	http://mistic.leloir.org.ar/results.php?jobid=20170611947015544
CD01424	MGS_CPS_II	http://mistic.leloir.org.ar/results.php?jobid=20170611337071057
CD03278	ATP-binding cassette domain of barmotin	http://mistic.leloir.org.ar/results.php?jobid=20170611948108735
CD03481	Transducer domain	http://mistic.leloir.org.ar/results.php?jobid=2017061194942951
CD01357	Aspartase	http://mistic.leloir.org.ar/results.php?jobid=20170611950419609
CD00036	Chitin/cellulose binding domains	http://mistic.leloir.org.ar/results.php?jobid=20170614134138131
CD00089	Protein kinase C-related kinase homology region 1 (HR1)	http://mistic.leloir.org.ar/results.php?jobid=20170614138375373
CD04371	DEP domain	http://mistic.leloir.org.ar/results.php?jobid=20170614139192179
CD00052	Eps15 homology domain	http://mistic.leloir.org.ar/results.php?jobid=20170614140306460
CD00173	Src homology 2 (SH2) domain	http://mistic.leloir.org.ar/results.php?jobid=20170614142492212
CD01926	cyclophilin_ABH_like domain	http://mistic.leloir.org.ar/results.php?jobid=201706111058271452
CD04912	ACT domains located C-terminal	http://mistic.leloir.org.ar/results.php?jobid=20170611105511638
CD00164	Ribosomal protein S1-like RNA-binding domain	http://mistic.leloir.org.ar/results.php?jobid=20170614204299821
CD01714	The electron transfer flavoprotein (ETF)	http://mistic.leloir.org.ar/results.php?jobid=201706111053598165
CD00585	Peptidase C1B subfamily	http://mistic.leloir.org.ar/results.php?jobid=201706111052469189
CD04867	TGS domain-containing YchF GTP-binding protein	http://mistic.leloir.org.ar/results.php?jobid=201706111051354847
CD02014	Thiamine pyrophosphate (TPP) family	http://mistic.leloir.org.ar/results.php?jobid=20170611953291860
