# Supplementary figures and images for: Protein sites with more coevolutionary connections tend to evolve slower, while more variable protein families acquire higher coevolutionary connections
Source: F1000Res. 2017 Jul 7;6:453. Originally published 2017 Apr 10. [Version 2] doi: 10.12688/f1000research.11251.2 (PMC5506539; doi:10.12688/f1000research.11251.2)

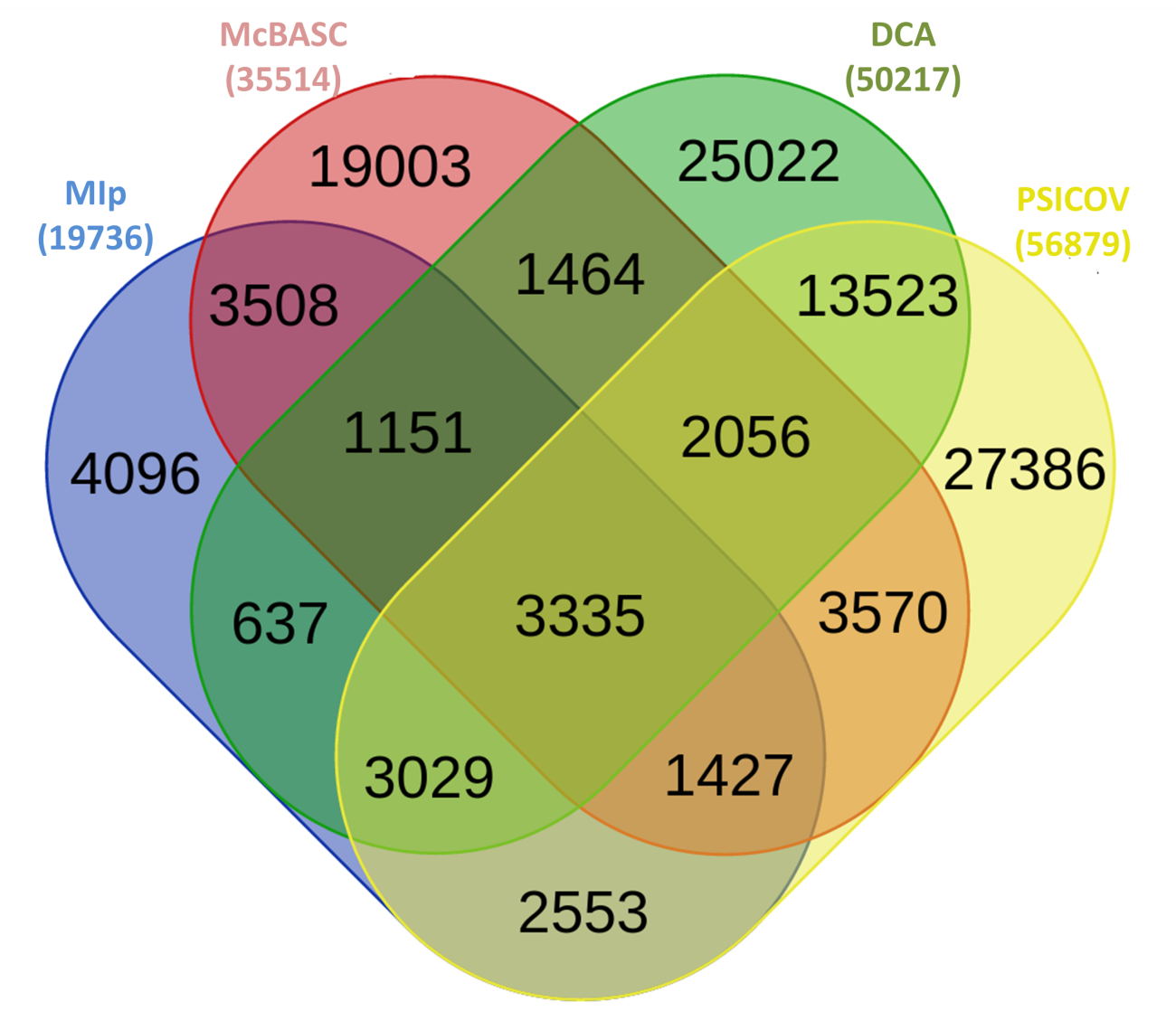

Supplement: Supplementary file 7 [file f1000research-6-13045-s0006.tgz › 732f1eea-8a33-48b9-9e6c-98706b727644.png]

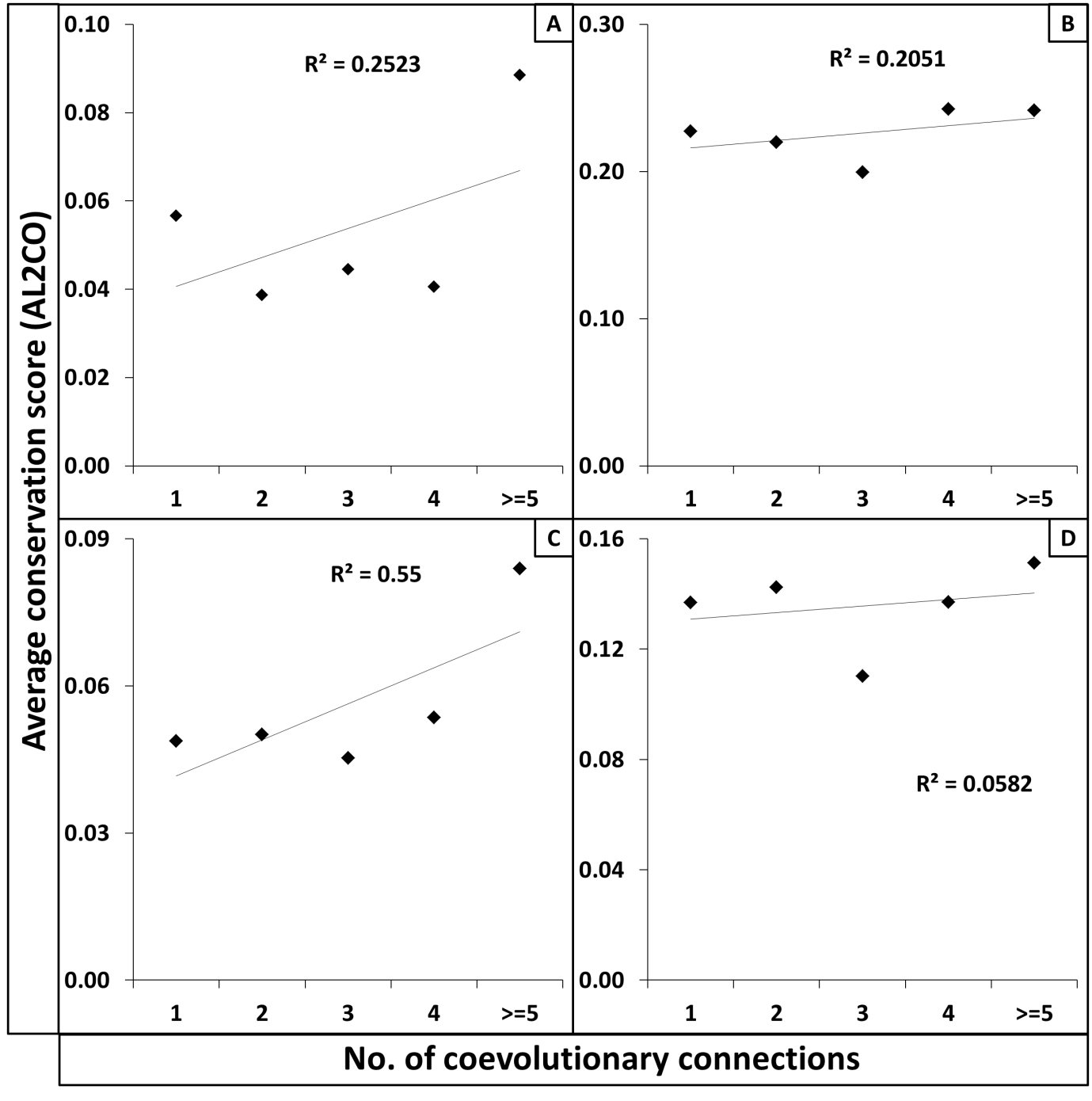

Supplement: Supplementary file 8 [file f1000research-6-13045-s0007.tgz › dfa9ba8d-41b5-4c0c-8b08-c20149baefd6.tif]

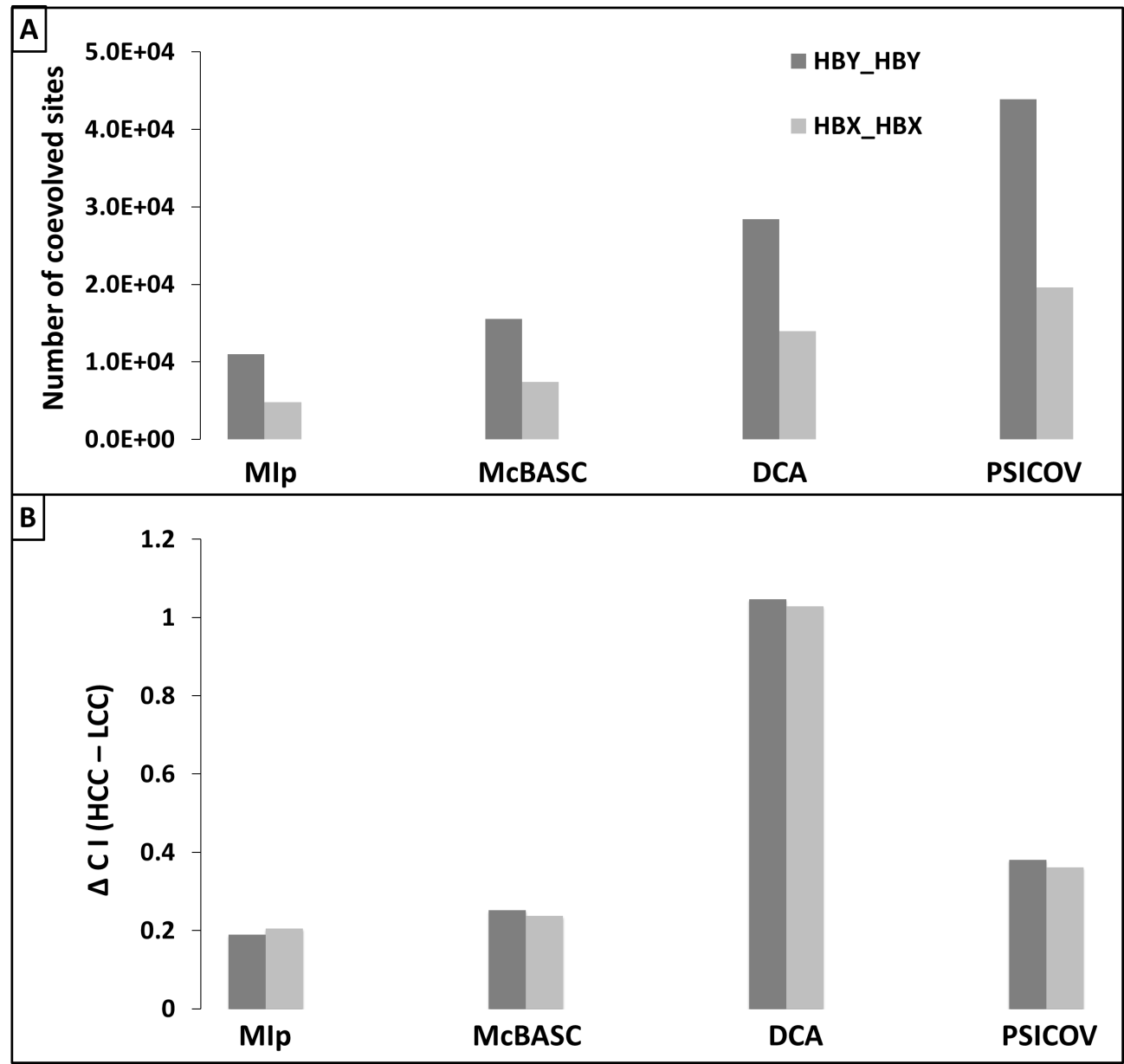

Supplement: Supplementary file 9 [file f1000research-6-13045-s0008.tgz › 4bfd0939-6d5c-4b4f-949d-db614f0d36e5.png]

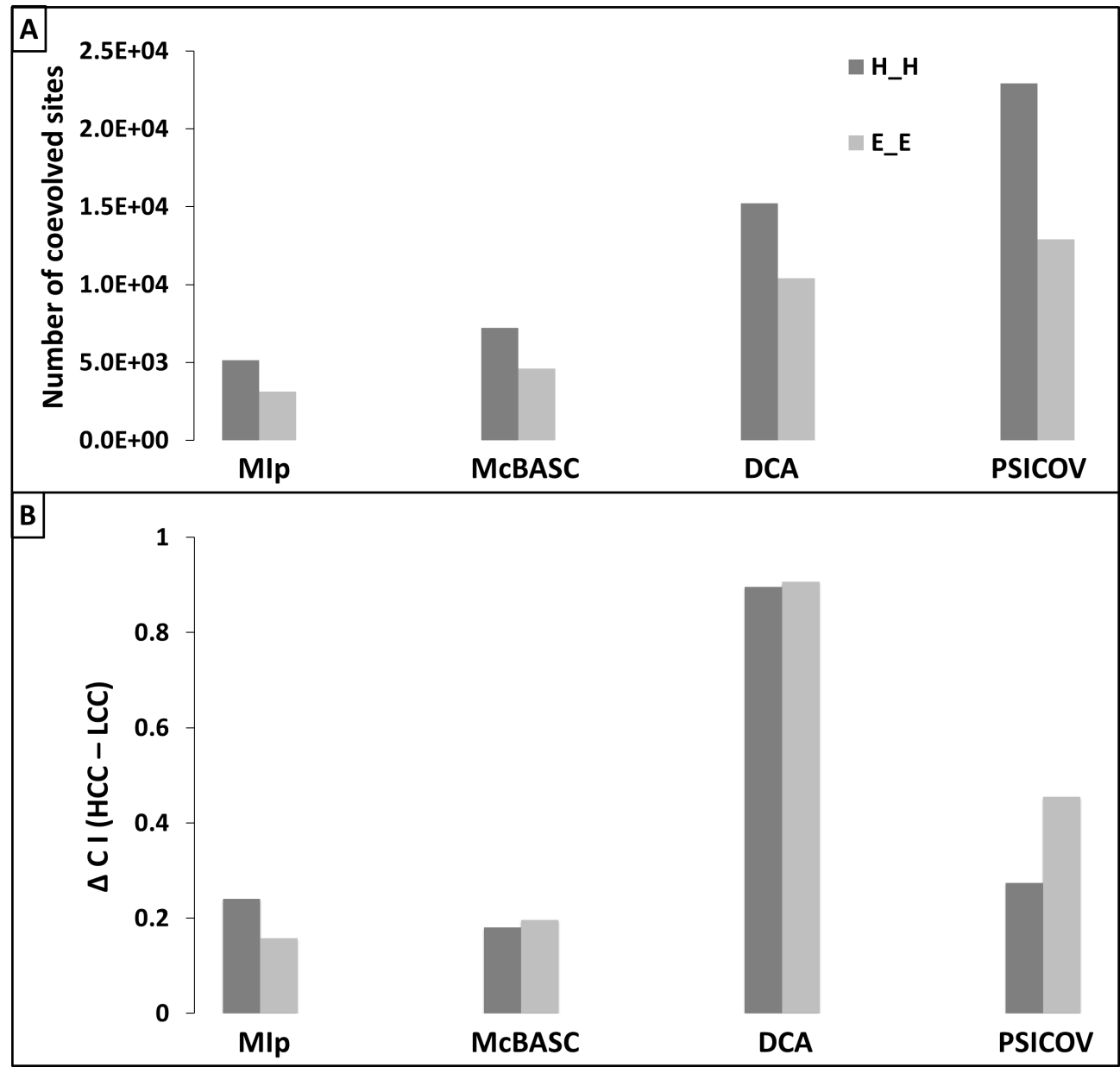

Supplement: Supplementary file 10 [file f1000research-6-13045-s0009.tgz › dfc35c4c-0ac1-47af-997b-3a7411e7f15d.png]

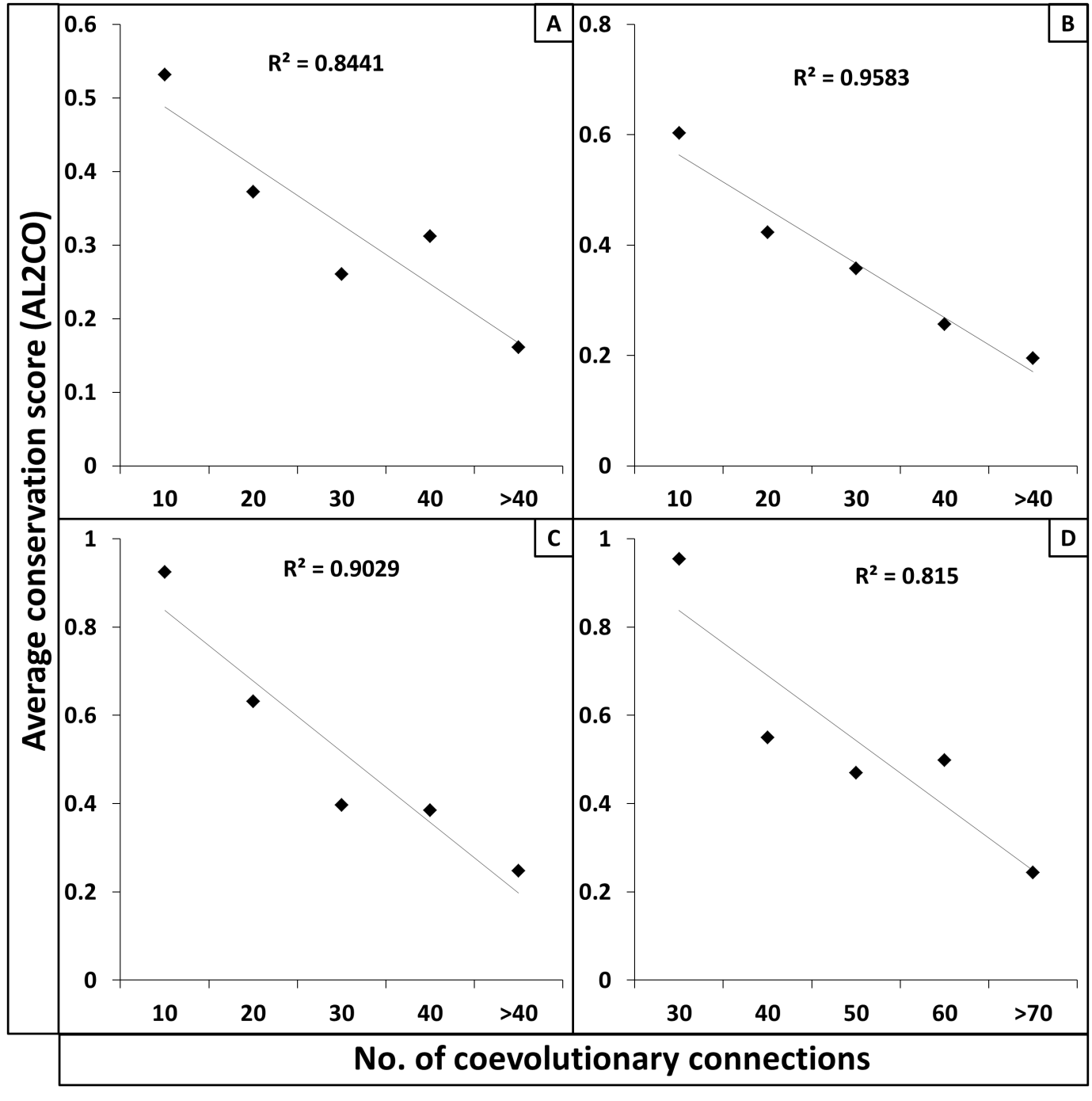

Supplement: Supplementary file 11 [file f1000research-6-13045-s0010.tgz › 45ec3866-2f67-4e00-b176-20073c4ac9f5.tif]

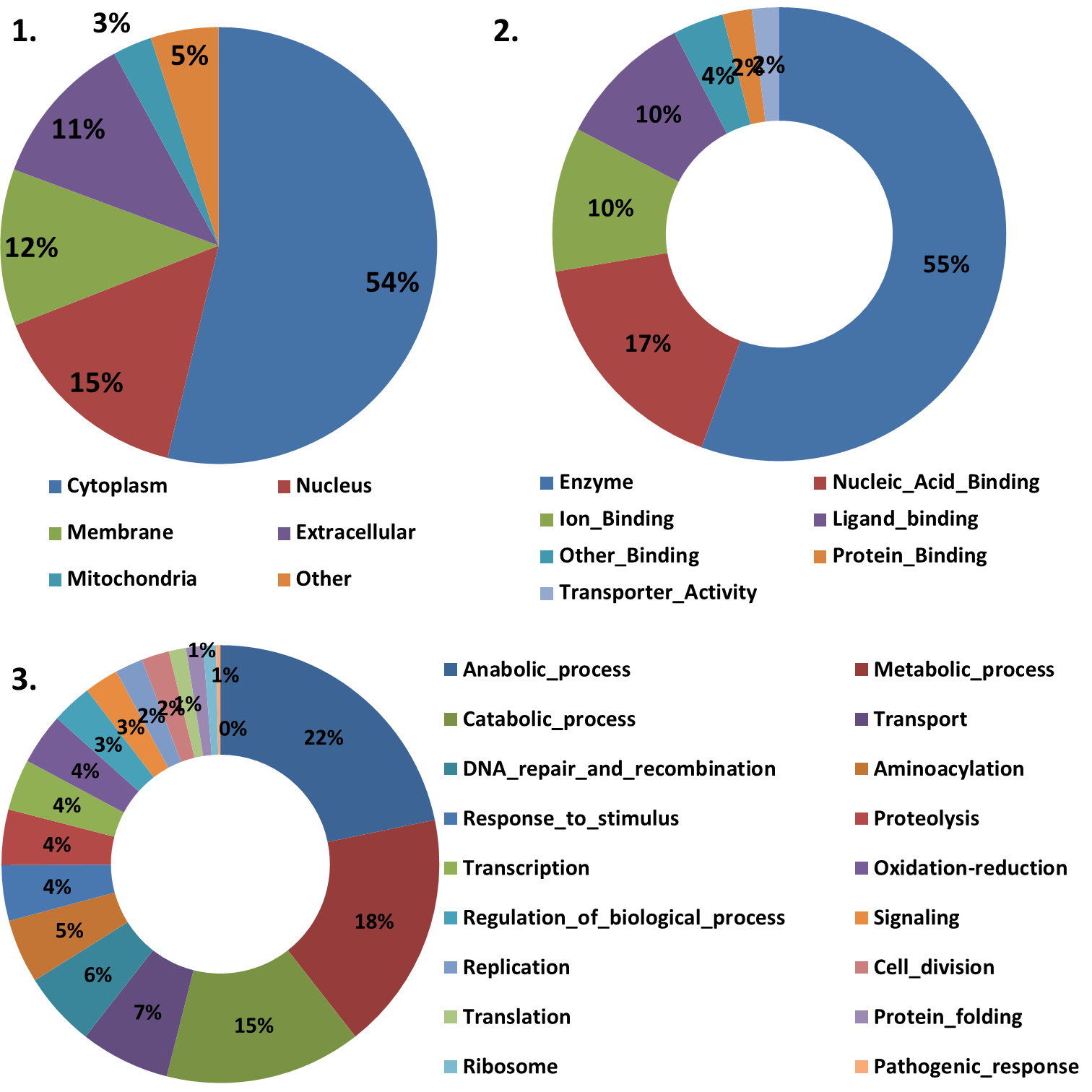

Supplement: Supplementary file 12 [file f1000research-6-13045-s0011.tgz › 2e67706f-63ba-457b-8378-48962e86476e.png]

A.

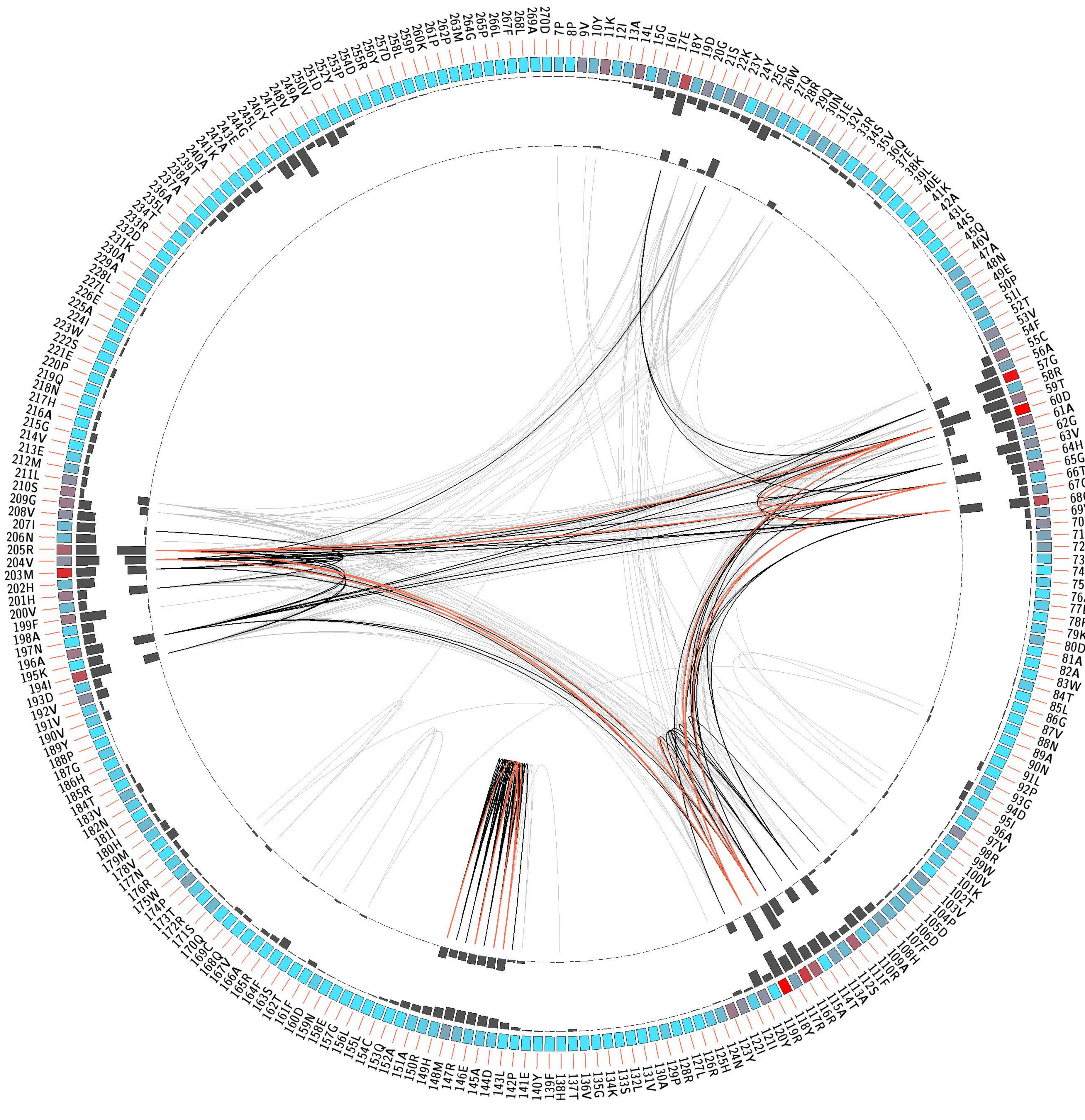

B.

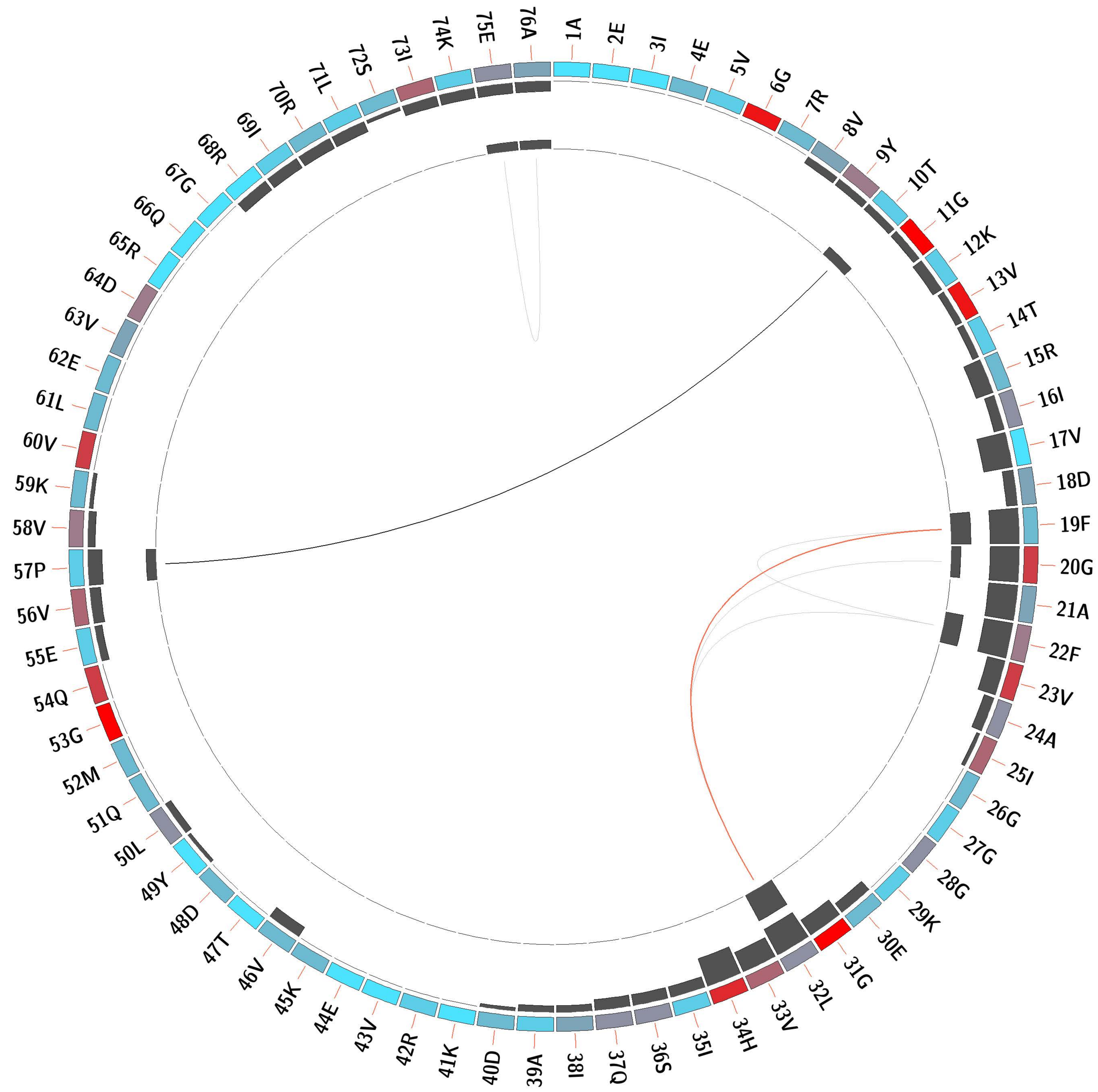

Supplement: Supplementary file 13 [file f1000research-6-13045-s0012.tgz › 48726ca7-10e7-4041-93e6-2add87843f66.pdf]
