# Supplementary material for: Protein sites with more coevolutionary connections tend to evolve slower, while more variable protein families acquire higher coevolutionary connections
Source: F1000Res. 2017 Jul 7;6:453. Originally published 2017 Apr 10. [Version 2] doi: 10.12688/f1000research.11251.2 (PMC5506539; doi:10.12688/f1000research.11251.2)
Supplement: Peer review history

# Peer review: Referee response for version 2

**DOI:** 10.5256/f1000research.13045.r24094

**Published:** 2017-07-10

**Authors:** Anna Panchenko

**Affiliations:** 1 Computational Biology Branch, National Institutes of Health, Bethesda, MD, USA

The authors have addressed my comments.

I have read this submission. I believe that I have an appropriate level of expertise to confirm that it is of an acceptable scientific standard.

# Peer review: Referee response for version 1

**DOI:** 10.5256/f1000research.12138.r21734

**Published:** 2017-05-03

**Authors:** Ramanathan Sowdhamini

**Affiliations:** 1 National Centre for Biological Sciences, Tata Institute of Fundamental Research, Bangalore, Karnataka, India

The evolutionary conservation of a large number of predicted co-evolving residue pairs has been investigated for possible correlation between the extent of conservation and the strength of co-evolving residue networks. Co-evolution has been predicted by four different popular algorithms. Residues with a high networking of co-evolved residues are found to be more evolutionarily conserved. However, the same trend is not true at the entire protein domain family level and evolutionarily conserved protein families appear to exhibit less co-evolved network of residues. Likewise, solvent-accessible residues were predicted to retain more co-evolutionary connections in comparison to solvent-buried residues. These are interesting observations, but the connections between these individual observations and possible implications/applications will be good to include in the paper.

 Queries:

 The first sentence in the Abstract could be changed to "Amino acid exchanges within proteins sometimes compensate for one another and could therefore be co-evolved." since this fact of tight linking is not well-known and forms one of the questions in this study.

 Page 4: It will be nice to explain how the conservation score (within the AL2CO program) is calculated.

 Was there no check for consensus in predicting the co-evolved residues? For instance, to see whichever are predicted by three or more methods … It will be interesting to examine the results for subset of such highly predicted co-evolved sites.

 Page 4: “Representative three-dimensional (3D) structures were collected

 for each family from the Protein Data Bank” – to provide details as to how they were selected?

 Page 5: This statement “Observation of strong positive correlation between coevolutionary connections and evolutionary conservation within the coevolved sites selected based on structural proximity suggests that highly coevolved protein sites tend to evolve slower.” seems to be apparently counter-intuitive. How can highly conserved sites be co-evolving also? Highly conserved sites usually imply high degree of identity (self-amino acid preservation). If so, how a co-evolutionary index can be set up for two spatially proximate residues which remain identical? Please explain for the benefit of the readers.

I have read this submission. I believe that I have an appropriate level of expertise to confirm that it is of an acceptable scientific standard.

# Response

**Published:** 2017-06-14

**Authors:** Saikat Chakrabarti

**Affiliations:** Indian Institute of Chemical Biology, India

**The evolutionary conservation of a large number of predicted co-evolving residue pairs has been investigated for possible correlation between the extent of conservation and the strength of co-evolving residue networks. Co-evolution has been predicted by four different popular algorithms. Residues with a high networking of co-evolved residues are found to be more evolutionarily conserved. However, the same trend is not true at the entire protein domain family level and evolutionarily conserved protein families appear to exhibit less co-evolved network of residues. Likewise, solvent-accessible residues were predicted to retain more co-evolutionary connections in comparison to solvent-buried residues. These are interesting observations, but the connections between these individual observations and possible implications/applications will be good to include in the paper.**

We thank the reviewer for the positive comments. Current study does not provide practical application in its current form, but does offer insight into the underlying properties of covariation/coevoluution methods and the relationship of these methods with evolutionary rate. However, the knowledge regarding the intricate relationship between evolutionary variability and coevolutionary connection is very important to gain insight about the dynamics and pattern of evolutionary history of protein families. The variable nature of this intricate balance is perhaps crucial in determining the overall conservation and/or flexibility of functionally important sites within certain protein families.  

**The first sentence in the Abstract could be changed to "Amino acid exchanges within proteins sometimes compensate for one another and could therefore be co-evolved." since this fact of tight linking is not well-known and forms one of the questions in this study.**

We thank the reviewer for her thoughtful comment. We agree to change the line in the abstract.

**Page 4: It will be nice to explain how the conservation score (within the AL2CO program) is calculated.**

Information on conservation score calculation is provided in manuscript subsection “Calculation of amino acid conservation”. The AL2CO (manuscript reference: 34) program performs conservation analysis in a comprehensive and systematic way. We used independent count (sequence weighting scheme) and matrix based sum-of-pair (conservation calculation method) measure scoring scheme of AL2CO program to calculate evolutionary conservation of each coevolved sites or column in the alignment. These scoring functions use the summation of the products of frequencies for the column for every combination of amino acid *a* and *b* and multiplies the products by the corresponding BLOSUM62 matrix amino acid substitution frequencies.

**Was there no check for consensus in predicting the co-evolved residues? For instance, to see whichever are predicted by three or more methods … It will be interesting to examine the results for subset of such highly predicted co-evolved sites.**

We thank the reviewer for her insightful opinion. Figure S1 in supplementary file 1 shows number of coevolved pairs predicted (in blue, pink, green and yellow) by different programs (MIp, MCBASC, DCA and PSICOV, respectively) and common pairs (in black) between them. We have performed correlation analysis on consensus data (for ex. 3335 coevolved pairs predicted by all four programs), we have observed similar trend, but as the number of consensus predicted coevolved sites are not so large, we have not provided in the results.

  

**Page 4: “Representative three-dimensional (3D) structures were collected**

**for each family from the Protein Data Bank” – to provide details as to how they were selected?**

We have selected 3D structure of each family from the conserved domain database (CDD) alignment (represented as first sequence in alignment file).

**Page 5: This statement “Observation of strong positive correlation between coevolutionary connections and evolutionary conservation within the coevolved sites selected based on structural proximity suggests that highly coevolved protein sites tend to evolve slower.” seems to be apparently counter-intuitive. How can highly conserved sites be co-evolving also? Highly conserved sites usually imply high degree of identity (self-amino acid preservation). If so, how a co-evolutionary index can be set up for two spatially proximate residues which remain identical? Please explain for the benefit of the readers.**

We thank the reviewer for these useful comments. We agree with the apparent counter intuitiveness of the sentence. However, it is the fact that we observed. The obvious question is why such apparently contrasting relationship exists. Perhaps, both coevolutionary and evolutionary changes are dynamic processes and for a given protein site at a certain point of evolutionary conservation status, highest coevolutionary connections are observed. This evolutionary conservation status of the site is perhaps selected and maintained. One probable explanation could be that these highly coevolved sites might act as ‘coevolutionary hub’ and therefore changes at these sites would affect many other connected sites. However, we must mention that in this study the higher conservation is with respect to other coevolving sites and not necessarily meant completely conserved sites.

# Peer review: Referee response for version 1

**DOI:** 10.5256/f1000research.12138.r21733

**Published:** 2017-04-25

**Authors:** Anna Panchenko

**Affiliations:** 1 Computational Biology Branch, National Institutes of Health, Bethesda, MD, USA

This paper expands on a previous study (Ref 32) and shows that the number of inter-residue coevolutionary relationships can be correlated with the evolutionary conservation of a protein site and protein family. The authors applied four different algorithms to calculate the coevolutionary relationships between sites and an overall trend observed in this study is confirmed by different methods. Interestingly, the absolute scale of site conservation for sites with the same number of coevolutionary relationships can differ drastically between methods (for example McBASC and MIp on Figure 1). I wonder if the sets of pairwise correlated sites overlap between different methods. I would also suggest using MISTIC server which can provide information on conservation, coevolution and structure mapping. The relationships between coevolution and diversity of protein families is interesting and intriguing, can it be related to the quality of alignments, one of the major factors defining the accuracy of coevolutionary detection algorithms? It is important also to discuss the difference between covariation and coevolution, the latter is not necessarily the cause, see some recent studies: PMID:25944916).

I have read this submission. I believe that I have an appropriate level of expertise to confirm that it is of an acceptable scientific standard, however I have significant reservations, as outlined above.

# Response

**Published:** 2017-06-14

**Authors:** Saikat Chakrabarti

**Affiliations:** Indian Institute of Chemical Biology, India

**This paper expands on a previous study (Ref 32) and shows that the number of inter-residue coevolutionary relationships can be correlated with the evolutionary conservation of a protein site and protein family. The authors applied four different algorithms to calculate the coevolutionary relationships between sites and an overall trend observed in this study is confirmed by different methods. Interestingly, the absolute scale of site conservation for sites with the same number of coevolutionary relationships can differ drastically between methods (for example McBASC and MIp on Figure 1).**

We thank the reviewer for the positive comments. We agree with reviewer's comment, the observed scale of values obtained from multiple coevolutionary programs varies a lot. The probable reason for such observation can be the algorithm used by individual programs for calculation of covariation/coevolution. We are providing our point-by-point response in the following.

**I wonder if the sets of pairwise correlated sites overlap between different methods.**

Figure S1 in supplementary file 1 shows number of coevolved pairs predicted (in blue, pink, green and yellow) by different programs (MIp, MCBASC, DCA and PSICOV, respectively) and common pairs (in black) between them.

**I would also suggest using MISTIC server which can provide information on conservation, coevolution and structure mapping.**

We thank the reviewer for the comment. MISTIC ( mutual information server to infer coevolution) is an online server, hence running for large number of protein families is not feasible. However, as case studies, we have performed the analysis on MISTIC server for 20 protein families (including CD01291 and CD00164 families provided in the paper). Result of MISTIC server for CD01291 is available at http://mistic.leloir.org.ar/results.php?jobid=201705252335594338 and for CD00164 at http://mistic.leloir.org.ar/results.php?jobid=201705269510226.

Circos representation of result for both the families is as follows:

CD01291 (Pseudouridine synthases family):

Link:http://mistic.leloir.org.ar/Results/job201705252335594338/circos/circos201705252335594338.png

CD00164 (Ribosomal protein S1-like RNA-binding domain):

Link: http://mistic.leloir.org.ar/Results/job201705269510226/circos/circos201705269510226.png

MI Circo is a sequential circular representation of the MSA and the information it contains. Coloured square boxes of the second circle indicate the MSA position conservation (highly conserved positions are in red, while less conserved ones are in blue).

Lines connect pairs of positions with MI greater than 6.5 (Marino Buslje *et al*, 2009). **Red** edges represent the top 5%, **black** ones are between 70% and 95%, and **gray** edges account for the remaining 70%.

Interestingly observed coevolutionary network predicted for both the families are similar to our study. Where CD01291 family has higher coevolutionary network connections for fewer variables sites whereas CD00164 family has less coevolutionary connections and overall less conserved.

Result for other families:

CD01424 (MGS_CPS_II):

http://mistic.leloir.org.ar/results.php?jobid=20170611337071057

CD01887 (Initiation Factor 2 (IF2):

http://mistic.leloir.org.ar/results.php?jobid=20170611409092676

CD03377 (Thiamine pyrophosphate (TPP family):

http://mistic.leloir.org.ar/results.php?jobid=20170611947015544

CD03278 (ATP-binding cassette domain of barmotin):

http://mistic.leloir.org.ar/results.php?jobid=20170611948108735

CD03481 (Transducer domain):

http://mistic.leloir.org.ar/results.php?jobid=2017061194942951

CD01357 (Aspartase):

http://mistic.leloir.org.ar/results.php?jobid=20170611950419609

CD00036 (Chitin/cellulose binding domains):

http://mistic.leloir.org.ar/results.php?jobid=20170614134138131

CD00089 (Protein kinase C-related kinase homology region 1 (HR1)):

http://mistic.leloir.org.ar/results.php?jobid=20170614138375373

CD04371 (DEP domain):

http://mistic.leloir.org.ar/results.php?jobid=20170614139192179

CD00052 (Eps15 homology domain):

http://mistic.leloir.org.ar/results.php?jobid=20170614140306460

CD00173 (Src homology 2 (SH2) domain):

http://mistic.leloir.org.ar/results.php?jobid=20170614142492212

CD01926 (cyclophilin_ABH_like domain):

http://mistic.leloir.org.ar/results.php?jobid=201706111058271452

CD04912 (ACT domains located C-terminal):

http://mistic.leloir.org.ar/results.php?jobid=20170611105511638

CD00164 (Ribosomal protein S1-like RNA-binding domain):

http://mistic.leloir.org.ar/results.php?jobid=20170614204299821

CD01714 (The electron transfer flavoprotein (ETF)):

http://mistic.leloir.org.ar/results.php?jobid=201706111053598165

CD00585 (Peptidase C1B subfamily):

http://mistic.leloir.org.ar/results.php?jobid=201706111052469189

CD04867 (TGS domain-containing YchF GTP-binding protein):

http://mistic.leloir.org.ar/results.php?jobid=201706111051354847

CD02014 (Thiamine pyrophosphate (TPP) family):

http://mistic.leloir.org.ar/results.php?jobid=20170611953291860

**The relationships between coevolution and diversity of protein families is interesting and intriguing, can it be related to the quality of alignments, one of the major factors defining the accuracy of coevolutionary detection algorithms?**

We thank the reviewer for bringing very important point of quality of alignment for coevolutionary analysis. Quality of alignment is major factor in the analysis and for this reason we have utilized manually curated CDD alignments.  

**It is important also to discuss the difference between covariation and coevolution, the latter is not necessarily the cause, see some recent studies: PMID: 25944916).**

We thank the reviewer for providing the information. In this study we have not checked/compared the difference between the two concepts of covariation and coevolution. We have used different programs (MIp, McBASC, DCA and PSICOV) which calculate covariation among protein sites in tree-independent manner. In this study, it was assumed that observed patterns of covariation are caused by molecular coevolution and they were treated synonymously.

# Response

**Published:** 2017-06-15

**Authors:** Anna Panchenko

**Affiliations:** National Library of Medicine & NIH, USA

I would like thank the authors for adequately responding to my comments.
